# Insights from *Bacillus anthracis* strains isolated from permafrost in the tundra zone of Russia

**DOI:** 10.1371/journal.pone.0209140

**Published:** 2019-05-22

**Authors:** Vitalii Timofeev, Irina Bahtejeva, Raisa Mironova, Galina Titareva, Igor Lev, David Christiany, Alexander Borzilov, Alexander Bogun, Gilles Vergnaud

**Affiliations:** 1 State Research Center for Applied Microbiology & Biotechnology (FBIS SRCAMB), Obolensk, Russia; 2 Institute for Integrative Biology of the Cell (I2BC), CEA, CNRS, Univ. Paris‐Sud, Université Paris‐Saclay, Gif‐sur‐Yvette cedex, France; Institut National de la Recherche Agronomique, FRANCE

## Abstract

This article describes *Bacillus anthracis* strains isolated during an outbreak of anthrax on the Yamal Peninsula in the summer of 2016 and independently in Yakutia in 2015. A common feature of these strains is their conservation in permafrost, from which they were extracted either due to the thawing of permafrost (Yamal strains) or as the result of paleontological excavations (Yakut strains). All strains isolated on the Yamal share an identical genotype belonging to lineage B.Br.001/002, pointing to a common source of infection in a territory over 250 km in length. In contrast, during the excavations in Yakutia, three genetically different strains were recovered from a single pit. One strain belongs to B.Br.001/002, and whole genome sequence analysis showed that it is most closely related to the Yamal strains in spite of the remoteness of Yamal from Yakutia. The two other strains contribute to two different branches of A.Br.008/011, one of the remarkable polytomies described so far in the *B*. *anthracis* species. The geographic distribution of the strains belonging to A.Br.008/011 is suggesting that the polytomy emerged in the thirteenth century, in combination with the constitution of a unified Mongol empire extending from China to Eastern Europe. We propose an evolutionary model for *B*. *anthracis* recent evolution in which the B lineage spread throughout Eurasia and was subsequently replaced by the A lineage except in some geographically isolated areas.

## Introduction

The etiological agent of the anthrax disease is the gram-positive bacterium *Bacillus anthracis*. A key feature of this microorganism, which largely determines its epidemiological potential and population structure, is the ability to form endospores, extremely resistant to adverse environmental factors and able to remain viable for a long time [1–12]. High preservation of spores explains that disease outbreaks leading to significant economic damage, mass mortality of livestock, and human victims may occur in regions where this disease has not been observed for decades. In addition, due to the high virulence of *B*. *anthracis*, the stability of endospores in the environment and the simplicity of cultivation, this bacterium can be turned into a potential biological weapon or tool for bioterrorism [13, 14], as illustrated by the anthrax contaminated letters in 2001 in the USA [15].

Anthrax is now very rare in most European countries [16] but remains a significant problem mainly in sub-Saharan Africa and in some regions of Asia [17–20]. Anthrax is endemic in Russia, where the disease manifests itself as sporadic cases among animals and rare cases of the disease among the population [21]. The presence of large territories hosting populations of wild and domestic ungulates creates a favorable context for disease outbreaks of epizootics, and the low human population density in most parts of the country makes it difficult to conduct anti-epidemiс measures, and to correctly account for anthrax animal burial sites. Past animal burial sites are often not documented and occasionally corpses were not buried. These burial grounds, and entire territories where previous epizootics took place, may become involved in increased economic activities, which, given the potentially high preservation of *B*. *anthracis* spores in a cold environment, could lead to new outbreaks of the disease [22]. Of particular interest in this regard is the tundra zone of Russia, located between 55 and 68 degrees north latitude.

One of the features of this climatic zone is the presence of permafrost. Permafrost is defined as lithosphere material (soil and sediment) permanently exposed to temperatures ≤0°C and remaining frozen for at least two consecutive years. Permafrost can extend down to more than 1000 m in depth and remain frozen for thousands of years [23, 24]. In permafrost conditions, the preservation of microorganisms can significantly increase, and thus permafrost is a peculiar accumulator of microbiota [25, 26]. The preservation of spores of bacilli, including *B*. *anthracis*, in the permafrost theoretically should significantly exceed the preservation of microorganisms in the vegetative form. Consequently, permafrost might allow the discovery of archaic forms of this microorganism, which could supplement our knowledge of the evolution of the anthrax microbe. We investigated strains of *B*. *anthracis* isolated in two tundra zones—during the outbreak of anthrax in Yamal in the summer of 2016 and during extraction of paleontological material from the permafrost in Yakutia in 2015.

## Materials and methods

### Yamal samples

In the summer of 2016, an outbreak of anthrax occurred on the Yamal Peninsula. The previous outbreak of anthrax was registered in 1941 and the district was officially declared "anthrax-free" territory of the USSR since 1968. In 2007, compulsory vaccination of reindeer was abandoned. On July 16 2016, the United Duty Control Service of the Yamal District was informed of deer death by private reindeer herders. The deer's deaths began at the estuary of the Nerosaveyakha River near Lake Pisyoto. Reindeer herders reported that sick animals became sluggish, began to move slowly, then fell and quickly died. No ulcers or skin lesions could be detected. On July 17 and 18, the Veterinary Service of the Yamal-Nenets Autonomous Area arrived in the area for clinical examination of animals and autopsy. Pathological material was sent to the Tyumen Regional Veterinary Laboratory. An autopsy showed cardiac and pulmonary insufficiency and the preliminary diagnosis was death from a heat stroke as July 2016 had anomalously hot weather, with temperatures above 35°C. Complementary investigations by veterinarians sent to reindeer herders camps on July 19–29 led the Tyumen Regional Veterinary Laboratory to report a suspicion of *B*. *anthracis* on July 24 and to take prophylactic measures (vaccination and chemotherapy, restrictions on animal movements). Additional samples were sent to the All-Russian Scientific Research Institute of Veterinary Virology and Microbiology (ARSRIVVM) in Pokrov (Moscow region).

By this time, the disease was observed in three foci: Lake Pisyoto area, Novoportovskaya tundra, Evayakha River area. The outbreak sites were separated by distances up to 250 kilometers, including two water barriers—the Gulf of Ob (width from 30 to 80 km) and the Taz Estuary (25 km average width).

On July 25, a complete laboratory confirmation of presence of *B*. *anthracis* in samples taken from dead deer was obtained by ARSRIVVM. A pure culture of *B*. *anthracis* was isolated from one sample, this strain was called 5875. The Governor of Yamal-Nenets Autonomous District introduced a quarantine regime in the Yamal district. State Research Center for Applied Microbiology & Biotechnology (SRCAMB) and ARSRIVVM employees went to Salekhard to sample and to consult local sanitary and medical institutions. Specialists of the Stavropol Anti-Plague Institute arrived on July 26.

Starting on July 26, arrived experts organized a diagnostic laboratory in the Center for Hygiene and Epidemiology in the Yamal-Nenets Autonomous District. During the outbreak, samples from people potentially infected were investigated by this diagnostic laboratory. Medical authorities decided to hospitalize to Salekhard all children from the outbreak areas even without visible signs of the disease. People evacuation to temporary camps equipped by that time were started and preventive antibiotic therapy was applied. SRCAMB experts flew from Salekhard to the disease focus in the Lake Pisyoto area to survey the local population and to collect samples. Until this time, both the local population and veterinarians working in this outbreak area were skeptical about the possibility of anthrax, and favored the heat shock hypothesis as a number of other infections harmless for humans could have caused the death of animals weakened by heat. Furthermore simultaneously with the beginning of vaccination and antibiotic therapy, the temperature of the infection focus decreased sharply, so there were reasons to believe that the cessation of new cases of the disease resulted from the lowering of temperature.

The typical development of the disease was as follows: a seemingly healthy deer would become suddenly weak, unable to walk and forced to lay down a few hours later, and would die after a few additional hours. In most cases the nose was bleeding (sometimes the anus too), rigor mortis developed at the usual time.

SRCAMB experts took samples of soil, water, blood samples, ears, and lymph nodes of dead deer. The samples were delivered to Salekhard on July 27, and eventually to SRCAMB on July 28. On July 29, strain 5875 was sent from ARSRIVVM to SRCAMB. On the same day, SRCAMB received a strain isolated from a sick person (washed off from skin infection) subsequently called Yamal_12 and insects caught by veterinarians working in the outbreak area: nine *Scopeuma stercorarium* and four *Hydrotaea dentipes* from Salekhard. On August 13, SRCAMB received strain 6063 isolated in epidemic area by ARSRIVVM.

### Yakutia samples

On August 12, 2015, miners extracting mammoth tusks from the permafrost on the bank of the river Uyandina in the Abyisk ulus (district) of Yakutia 57 km from the district center Belaya Gora (“White Mountain”, latitude N68.564567, longitude E144.769827) found two ice-frozen kittens of the cave lion *Panthera leo spelaea*. The discovery was remarkable by its unprecedented degree of preservation, the animal’s wool and soft tissues were preserved. The bodies of the kittens were transferred to paleontologists. Some samples were taken to the nearest scientific institution, the Institute of Oil and Gas of the Siberian Branch of the Russian Academy of Sciences in Yakutsk (IPMR SB RAS), for microbiological examination. June 01, 2016 an unknown bacillus-like strain was isolated from soil sample (depth of sampling was not known) in the laboratory of geochemistry of caustobioliths of IPMR SB RAS and sent to the Institute of Genetics and Selections of Industrial Microorganisms (GosNIIgenetika) in Moscow for identification. On September 01, 2016, this strain was identified as *B*. *anthracis*. Since this institute is not authorized nor equipped to carry out work with pathogenic microorganisms, this initial culture was destroyed. On September 21 2016 by order of the Chief State Sanitary Doctor of Yakutia, soil samples were collected in the paleontological discovery point. Six separately packed glass jars with soil samples (200 g each), taken from a depth of one to six meters with an interval of one meter were received by SRCAMB in December 2016.

### Animal experiments

#### Ethics statement

All protocols for animal experiments were approved by the SRCAMB Bioethics Committee (Permits No: VP-2016/4 and VP-2016/5). They were performed in compliance with the NIH Animal Welfare Insurance #A5476-01 issued on 02/07/2007 and the European Union guidelines and regulations on handling, care, and protection of laboratory animals (http://ec.europa.eu/environment/chemicals/lab_animals/home_en.htm).

Animals were purchased from the Laboratory Animals Breeding Center, Shemyakin and Ovchinnikov Institute of Bioorganic Chemistry, Pushchino, Russia. They were housed in polycarbonate cages with space for comfortable movement (five mice per 484 cm^2^ cage or two to three guinea pigs per 864 cm^2^ cage) and easy access to food and water, under constant temperature (22°C ± 2°C) and humidity conditions (50% ± 10%) and a 12-hour light/12-hour dark cycle.

Approved protocols provided scientifically validated humane endpoints, including pre-set criteria for euthanasia of moribund animals by CO_2_ inhalation. Animals were euthanized when they became lethargic, dehydrated, moribund, unable to rise, or non-responsive to touch. Surviving animals were euthanized after the observation period.

#### Mice

Six-to-eight-weeks-old BALB/C mice of both genders, weighing 18–20g were used in all experiments. They were fed with Mouse Mixed Fodder PK-120 (Laboratorkorm, Moscow, Russia).

For virulence evaluation, mice were randomly divided in four groups of ten animals and infected subcutaneously by doses of 10^0^, 10^1^, 10^2^ and 10^3^ spores/animal. They were observed for 30 days after infection.

For bioassay, we used groups of three mice for each tested sample. Mice were inoculated subcutaneously in the inner part of the upper thigh with sample dispended in 0.3 ml of PBS. Animals were observed during ten days, after which surviving mice were euthanized. Dead and euthanized mice were necropsied, and spleen and liver samples were inoculated on Petri dishes with selective anthrax agar (SRCAMB, Obolensk, Russia).

#### Guinea pigs

Guinea pigs were used for evaluating the virulence of strains isolated during the Yamal epidemic. Five-to-seven-weeks-old animals of both genders, weighing 350–450g were fed Granuled Fodder KK-122 (Laboratorkorm, Moscow, Russia) during the entire experiment (30 days). They were randomly divided in three groups of five animals and inoculated subcutaneously in the inner part of the upper thigh by doses of 10^2^, 10^3^, 10^4^ spores/animal in 0.5 ml of PBS. They were observed during 15 days, after which surviving animals were euthanized. Spleen and liver samples of dead and euthanized guinea pigs were inoculated on Petri dishes with selective anthrax agar (SRCAMB, Obolensk, Russia).

### Insects samples

Insects samples were washed from alcohol with sterile PBS, ground in a mortar with sterile sand, and the resulting homogenate was used for sowing on solid nutrient media and for isolating the total DNA that was used for PCR.

### Bacterial culture

GRM agar, selective anthrax agar [27], yolk agar and blood agar (SRCAMB, Obolensk, Russia) were used for bacterial cultivation.

### DNA extraction and PCR analyses

DNA from field and clinical samples was isolated using «Reagent kit «K-Sorb» for DNA extraction on microcolumns» (Syntol, Moscow, Russia). DNA from bacterial cultures was isolated using GenElute Bacterial Genomic DNA Kit (Sigma-Aldrich, Moscow, Russia).

PCR amplifications were run on the CFX96 Real-Time PCR Detection System (Bio-Rad, Moscow, Russia). For Multiple Loci VNTR (Variable Number of Tandem Repeats) Analysis (MLVA) and canSNP genotyping, 2.5× PCRmix M-427 with SYBR-GreenI (Syntol, Moscow, Russia) were used. PCR primers were synthesized by Syntol, Russia.

PCR detection of *B*. *anthracis* DNA was performed using the «MULTI-FLU» Real-Time PCR-test kit (SRCAMB, Obolensk, Russia) and «OM-screen-anthrax-RT» (Syntol, Moscow, Russia).

MLVA was performed using primers listed in Thierry et al. [28]. Monoplex PCR products and a 20 bp ladder (Bio-Rad, USA) were electrophoresed at 100 V for four hours on a 32-cm length 3% agarose gel prepared in 0.5× TBE. The DNA fragments were visualized with ethidium bromide staining and ultraviolet (312 nm) using the Doc-Print gel documenting system and PhotoCaptMw software version 99.04 (Vilber Lourmat, Marne-la-Vallée, France). PCR products larger than 600 bp were reanalyzed on 2% agarose gel for better resolution and any remaining ambiguity was confirmed using Experion Automated Electrophoresis System (Bio-Rad, Moscow, Russia) and DNA sequencing. canSNP-genotyping was performed as described in [29].

### Whole genome SNP analysis

Yamal strains whole-genome sequencing was performed using the Ion Torrent PGM and associated Ion PGM Reagents 400 Kit and Ion 318 Chip Kit (Life Technologies, Moscow, Russia). Yakutia strains whole-genome sequencing was performed using the Illumina MiSeq instrument and Miseq Reagent Kit v3 (Albiogen, Moscow, Russia). DNA libraries were prepared using Nextera DNA Library Preparation Kit (Albiogen, Moscow, Russia). Sequence reads archives were deposited under study accession PRJEB30558 and can be recovered at https://www.ebi.ac.uk/ena/data/view/PRJEB30558. The eleven archives are numbered ERR3170358 to ERR3170368.

Additional sequence read archives were recovered from the European Nucleotide Archive (ENA) using the enaBrowserTools (https://github.com/enasequence/enaBrowserTools). Genome assemblies were downloaded from NCBI and *in silico* converted into 100 bp. sequence reads files with a 50x coverage using a homemade python script. Sequencing reads were mapped on reference genome *B*. *anthracis* Ames ancestor assembly GCA_000008445 using BioNumerics version 7.6.3. Because Ion Torrent sequence data suffers from a relatively high indels error rate as compared to Illumina data, we allowed indel-tolerant mapping (gapped alignment) for this type of data [30]. SNPs were called within BioNumerics using the strict closed dataset option. After removal of duplicates, 673 entries were kept in the present investigation including one *B*. *cereus* strain used as outgroup. All evaluated WGS archives are listed in S1 Table. Minimum spanning trees were produced using BioNumerics allowing the creation of hypothetical missing links. Aggregations of SNPs potentially reflecting homologous horizontal gene transfer events (recombination) were searched using Gubbins [31].

## Results

### Investigation of the Yamal samples

PCR-diagnostic of soil, water and necropsy samples showed that all samples (n = 23) except soil from the reindeer herding camp (n = 5) contained DNA of *B*. *anthracis*. Culture medium (GRM agar, selective anthrax medium, yolk agar or blood agar) was inoculated with materials from investigated samples. Typical, *B*. *anthracis*-like colonies grew from all PCR positive samples, none from PCR-negative samples. Observation of these cultures under the microscope showed the presence of chains of gram-positive bacillus coated with a capsule. All these bacillus-like colonies were PCR positive for *B*. *anthracis*. Some colonies of extraneous microflora grew from these samples.

All insect samples were PCR-negative, and no colonies of *B*. *anthracis* or extraneous microflora could be recovered. This negative result might be due to the use of ethanol for preservation of entomological specimens. Consequently, we were unable to confirm or disprove the potential role of bloodsucking insects in the spreading of the disease over long distances.

Soil from the place of death, soil from the camp, water from a nearby lake, cervical lymph node, blood from the neck region, blood discharge from the anus, and all the clinical samples were used in a bioassay. All the mice (except mice infected by soil from the camp) died with symptoms of anthrax on the second day after infection, their spleen and injection sites contained live anthrax bacteria as shown by inoculation on Petri dishes.

MLVA genotyping was applied in order to establish whether several genotypes circulated in the epidemic zone. We initially used loci vrrA, Bams03, Bams05, Bams22, Bams44, and vntr23 derived from the MLVA7 scheme proposed by Thierry et al. [28]. This set of loci allowed to genotype all isolates during one day with a high degree of reliability and proved to be very useful in conducting a preliminary epidemiological investigation, when it is required to minimize the time spent on analysis, down to hours. Subsequently the 25 loci described by Lista et al. [32] were applied. The same MLVA profile was observed in all strains isolated in the summer of 2016 on the Yamal, regardless of the place of isolation (Lake Pisyoto area, Novoportovskaya tundra, Evayakha River area) and of the institution by which the samples were analyzed (S2 Table). Querying the *Bacillus anthracis* v4_1 MLVA database at http://microbesgenotyping.i2bc.paris-saclay.fr [28] indicated that the Yamal strains belong to the B-clade [33].

The Yamal MLVA25 genotype differed from all genotypes present in the MLVA database at four loci or more among the 25 loci. canSNP-genotyping [29] assigned all Yamal strains to B.Br.001/002 lineage in agreement with the MLVA based assignment.

The finding of a single MLVA25 profile suggested that one strain circulated throughout the epidemic, possibly from a unique source of infection of humans and animals. No strains in the collections of SRCAMB and Stavropol Anti-Plague Institute (Reference Center for the control of Anthrax) showed the same MLVA25 profile. We selected four strains collected in Lake Pisyoto outbreak area for further work including whole genome sequencing (Table 1).

**Table 1:** *B*. *anthracis* strains isolated from Yamal outbreak field and clinical samples.

Strain name	Source
Yamal_2	Cervical lymph node of dead deer
Yamal_8	Water from a lake
Yamal_10	Soil near a dead deer
Yamal_12	Cutaneous carbuncle of a sick person

The strains listed in Table 1 were typical for a combination of phenotypic properties, but the clinical isolate Yamal_12 initially had lecithinase activity, unlike the other isolates. After two passages on solid media, this activity was lost. All selected isolates were tested for virulence *in vivo*. Considering that the work was carried out during an emergency, and that it was extremely important to obtain the most complete and reliable data on the strains under study, guinea pigs were used in addition to the mouse model. All isolates were virulent and no statistically significant differences in their virulence were detected (S3 Table).

### Investigation of the Yakutia samples

Several colonies were cultivated on selective anthrax agar from the soil samples collected at a depth of two, three and four meters. In contrast, no colonies were recovered from soil samples collected at depth of one, five and six meters. The recovered colonies looked like *B*. *anthracis* colonies, did not show lecithinase and hemolytic activity, and were sensitive to the Gamma phage.

PCR analysis identified the colonies as *B*. *anthracis*. MLVA typing using seventeen loci (MLVA17) distinguished three genotypes, subsequently called 3Ya, 4Ya, 5Ya (S2 Table). Genotype 4Ya was equally represented in samples from a depth of two and three meters (Table 2). Genotypes 3Ya and 4Ya differed at four loci. Querying the *B*. *anthracis* MLVA database indicated that both are closest to strains belonging to canSNP clade A.Br.008/009. MLVA17 genotype 5Ya was identical to the Yamal MLVA17 genotype. These strains were virulent for mice but no statistically significant differences in their virulence were detected (see S3 Table)

**Table 2:** Yakutia soil samples investigations and strains selected for whole genome sequencing.

Depth	Detected MLVA17 genotypes	Representative strain_genotype name
1m	NA*	NA*
2m	3Ya, 4Ya	LP50_3Ya
3m	4Ya	LP51_4Ya
4m	5Ya	LP53_5Ya
5m	NA*	NA*
6m	NA*	NA*

*NA—not applicable

### Whole genome SNP analysis

Surprisingly, the recovered MLVA genotypes were very similar to already known MLVA genotypes. Furthermore, the Yamal MLVA genotype were identical to one of the three Yakut strains genotypes. Finally, we recovered three different MLVA genotypes from the same spot in Yakutia, whereas the Yamal 2016 outbreak was associated with a unique genotype in spite of its wide geographic dispersion. One simple explanation for this observation from the Yakut excavation could be contamination when analyzing the samples in SRCAMB. The resistance of the endospores is a well-known cause of accidental laboratory contaminations as recently recalled [34]. In order to investigate this possibility we sequenced four Yamal (Table 1) and three Yakut (Table 2) strains, together with the four *B*. *anthracis* strains in the SRCAMB collection showing the closest MLVA genotype. We performed a whole genome SNP analysis comparison of these eleven genomes with publicly available whole genome sequence data from 672 *B*. *anthracis* strains (S1 Table). S1 Fig. shows the relative position of the Yamal and Yakut strains within the global *B*. *anthracis* population represented by a selection of 42 *B*. *anthracis* strains. The four Yamal strains belong to B.Br001/002 in agreement with the canSNP typing. The Yakut LP53_Ya5 strain is the closest neighbor.

**Fig 1:**
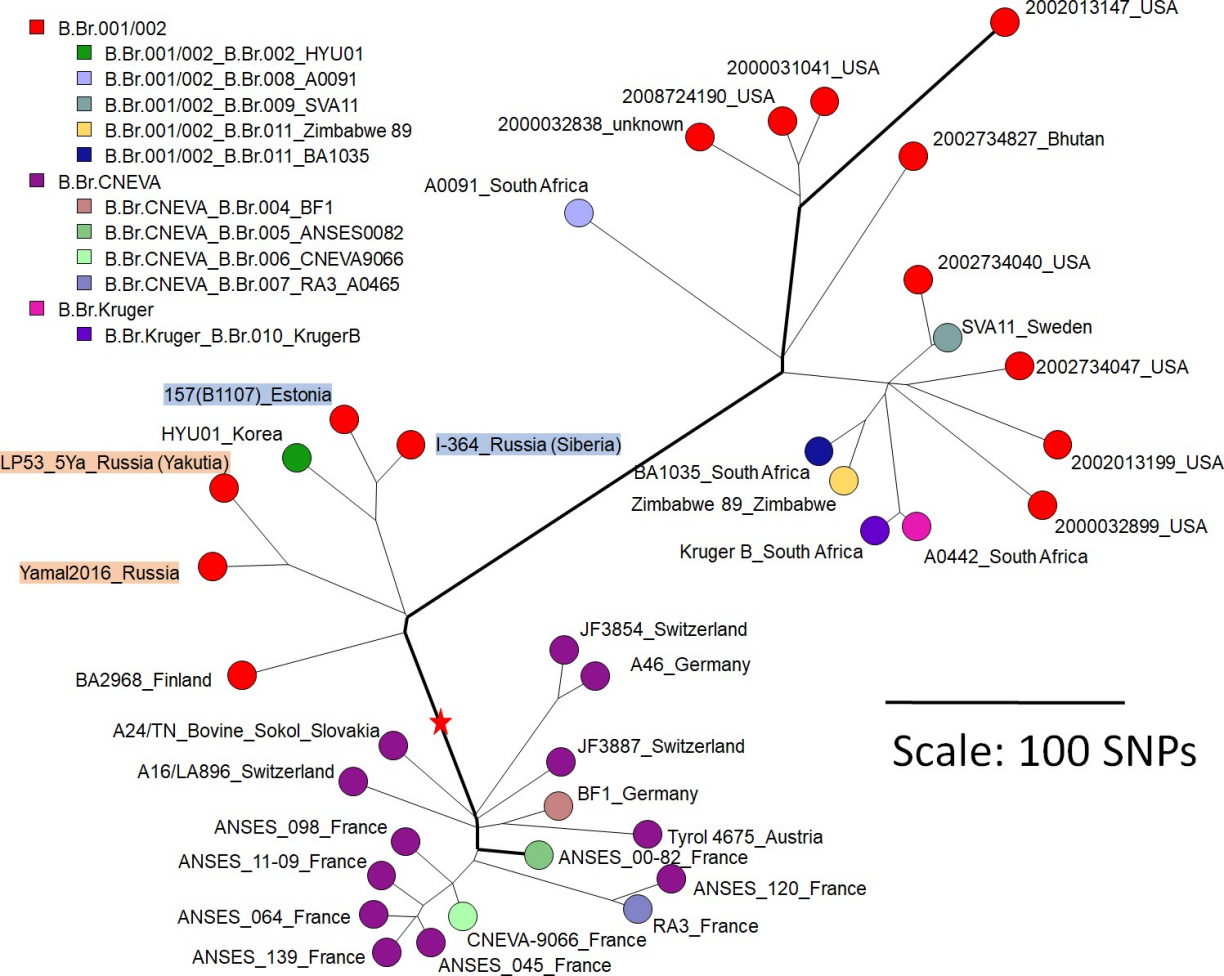
Position of the Yamal and LP53_5Ya Yakut *B*. *anthracis* strains within the B-clade. The Ames ancestor reference genome was used to root the minimum spanning tree (red star). Branch length is linearly proportional to the number of SNPs (scale bar). The minimum spanning tree is based on 2123 SNPs. The size of the resulting tree is 2125 indicating a very low level of homoplasia in agreement with previous reports. Each circle represents one strain. The color code reflects the lineage within the B-clade. Strains previously investigated by Sahl *et al*. are given a specific color code and named according to [38]. The longest and shortest root-to-tip paths are shown with bold lines. Starting from the red star, the longest lineage is defined by 449 SNPs whereas the shortest is defined by 77 SNPs i.e. a ratio of 5.8. The tags include strain name and country of isolation. The Yamal, LP53_5Ya Yakut, and closest neighbors from the SRCAMB collection are indicated with colored tags. The list of strains and sequence data accession used to make the figure is provided in S1 Table.

Fig 1 is a close-up on the B-clade, separated in the canSNP lineages B.Br.CNEVA and B.Br.001/002 [33]. B.Br.CNEVA and B.Br.001/002 are very similar in terms of lineage expansion (branch length) except for the fast-evolving B.Br.001/002 sublineage observed in North-America, Bhutan, Sweden and South-Africa including sub-lineage B.Br.Kruger. From the root of the B-clade to the tip, the longest and shortest lineages are defined by 449 and 77 SNPs respectively, corresponding to a ratio of 5.8 in the average expansion rate. B.Br.CNEVA was observed in mountainous areas in Western Europe whereas B.Br.001/002 strains were observed in Yamal, Siberia, Estonia (this report), Korea [35] and Finland [36] in addition to the fast lineage geographic distribution. The Yamal and Yakut LP53_5Ya strains belong to the same sublineage but are clearly separated by 65 SNPs (83 SNPs when optimizing SNP detection by focusing on the Yamal-Yakut strains, S2 Fig). It is tempting to speculate that the fast evolving lineage including the “Kruger” clade is the result of a highly successful export to South Africa, possibly as a result of European trade along maritime routes in medieval times or later. Fast expansion may also reflect the presence of a hypermutator phenotype [37] and additional strains revealing new intermediate lineages will be needed to better understand the phylogeography of the rare B-clade.

**Fig 2:**
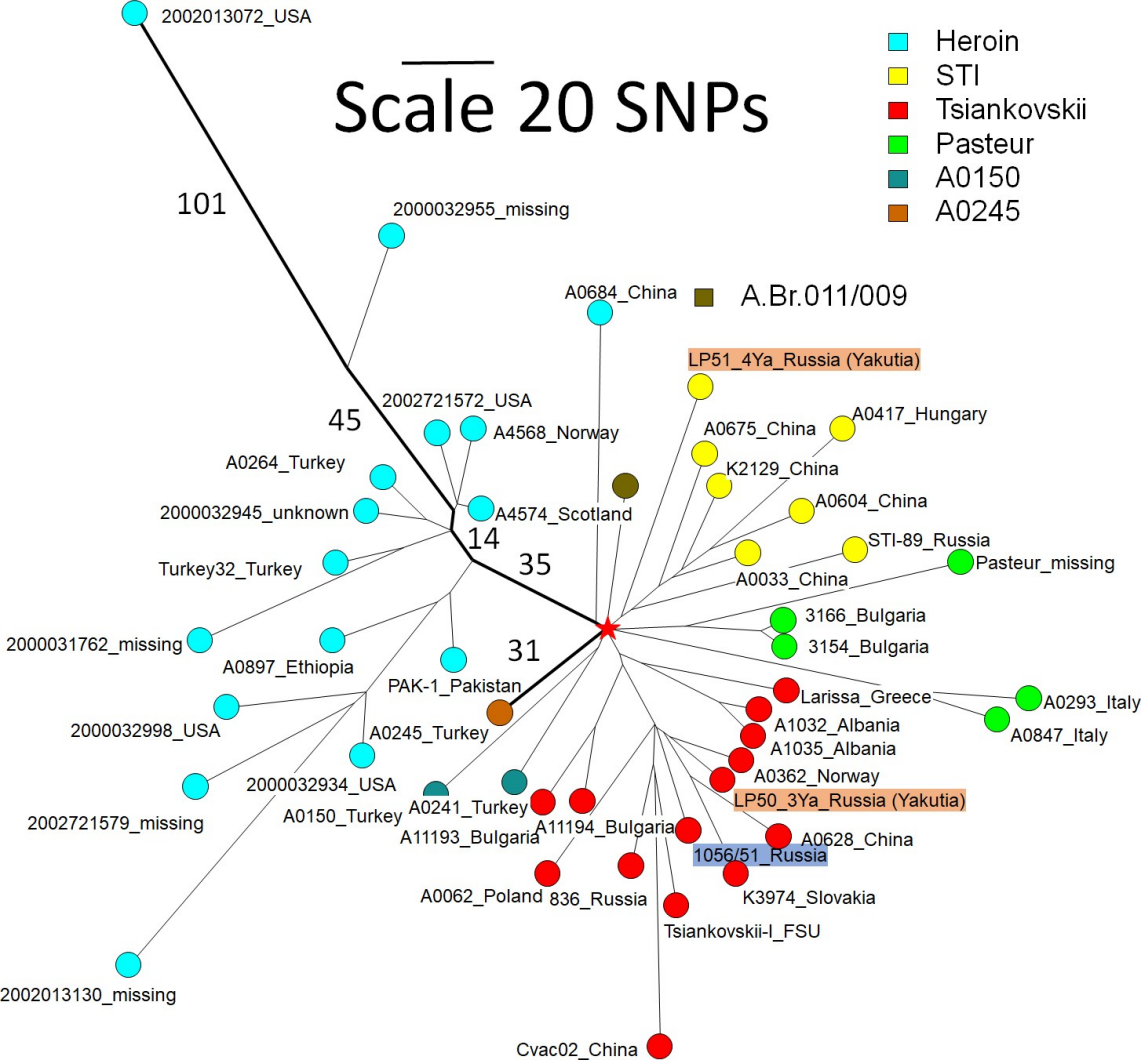
A.Br008/011 polytomy, minimum spanning tree, linear scale. The color coding reflects the lineages within the A.Br.008/011 polytomy using the previously defined classification [38]. Geographic origin is indicated when known. The root (red star) is defined using the Ames ancestor reference genome. One strain representing the A.Br011/009 lineage is included. Branch lengths along the longest and shortest root-to-tip paths (bold branches) are indicated. The tree is based upon 1825 SNPs, and the level of homoplasia is 0.7% (the size of the tree is 1838). The list of strains used to make the figure is provided in S1 Table.

The two other Yakut strains, LP50_3Ya3 and LP51_4Ya, belong to the Transeurasian radiation TEA 008/011 [38]. TEA 008/011 is a remarkable polytomy with a star-like radiation pattern comprising seven branches. Fig 2 displays a minimum spanning tree based on the whole genome SNPs detected among strains assigned to TEA 008/011. The branches are named as previously proposed [38]. Distances from root to tips differ by a ratio of 6.39 among the different lineages. The shortest branch with 31 SNPs is represented by strain A0245 from Turkey. The longest, with 198 SNPs, is observed in lineage “Heroin”. The most represented lineages are “Heroin” [39–41], “STI” and “Tsiankovskii” [38]. The “Heroin” lineage contains one early split, three SNPs from the core of the polytomy. One branch is present in China, whereas the other branch is more complex in terms of geographic origin as it was isolated in many countries and includes strains recovered from human patients infected via drug usage. The difference in length is not the result of horizontal gene transfer events, as the associated SNPs do not show evidence for clustering [31,42]. The geographic origin of the heroin-associated strains is uncertain. Afghanistan is a likely option if heroin contamination occurred as part of the drug production or initial packaging processes. Other candidate countries are Turkey and Pakistan represented by strains defined by short lineages, or additional neighbor countries not represented in current *B*. *anthracis* databases. The “STI” lineage contains three early-branching sublineages. The first is defined by the Yakut LP51_4Ya strain, the second is present mostly in China, and the third corresponds to the STI vaccine strain from Russia. The Tsiankovskii vaccine strain, the Sverdlosk 1979 strain [38], and the Yakutia LP50_3Ya strain which is closest to a strain from Norway belong to lineage “Tsiankovskii”. This lineage is widespread in Russia and Eastern Europe, including Greece, Albania, Bulgaria, Poland. Lineage “Pasteur” contains two early-branching sublineages, one found in Bulgaria and the other in Italy in addition to the Pasteur I vaccine strain. Lineages “A0150” and “A0245” named here according to the one or two strains defining them were observed in Turkey. The last lineage leads to TEA Br011 corresponding to the A.Br.011/009 polytomy observed at high frequency in France and Italy [43, 44, 45]. In summary, eleven early-branching lineages are detected within the seven-branches TEA 008/011 polytomy, nine of which with a strong geographic assignment, to Turkey (2), China (2), Russia (2, including Northern Yakutia, Siberia), Italy (2), Bulgaria and France. The root (red star) defined by the branching point of the Ames ancestor reference strain is located on lineage “Tsiankovskii” at a distance of one SNP from the center of the polytomy connecting the six other branches. This might suggest that Europe is the geographic origin of the A.Br.008/011 polytomy. However, this argument is weak and extensive sequencing of additional A.Br.008/011 strains will be needed to establish the geographic origin of the polytomy.

One representative from A.Br.011/009 was included in Fig 2. The root of the six branches A.Br.011/009 polytomy [43, 44] is located along this branch at a distance of six SNPs from the root of the A.Br.008/011 polytomy. The A.Br.011/009 polytomy emerged later than the A.Br.008/011 polytomy [43, 44].

## Discussion

### The Yamal 2016 anthrax outbreak and implications

Anthrax is endemic in most of Russia, including Yamal. At the beginning of the Yamal colonization by the Russian Empire in the 17–18 centuries, cases of this disease were reported. The first outbreak was recorded in 1760. From 1898 to 1931, 66 epizootics were described, during which it was estimated that more than a million deer died. In the 1940s, the whole reindeer livestock was vaccinated but in subsequent years, the number of vaccinated animals was lower. For example, in the 1960s, from 65 to 82% of the total number of deer was vaccinated, which proved sufficient to prevent epidemics. Thus, a centuries-old cycle of anthrax circulation in the tundra of the Far North was interrupted [46].

The absence of outbreaks suggested that the soil in the tundra was sanitized and no longer contained anthrax spores. In 1968, 360 soil samples from places of recorded mass death of reindeers were examined and no *B*. *anthracis* strains could be recovered [47]. This suggested that tundra soil conditions (pH 3–5, humus content lower than 3%) are unfavorable to maintain the viability of the spores. In 2007, deer vaccinating was stopped. During June-July of 2016, the air temperature in the Yamal epidemic area was 5–9 degrees higher than normal, and did not fall below 18 C. The soil reached a temperature of 25 C at a depth of 10 cm and 7 C at a depth of one meter. This was combined with a very small amount of rain precipitation [48]. Apparently such an anomalous warm climate led to the thawing of permafrost, and viable *B*. *anthracis* spores could be exposed to the surface [3, 49].

According to the testimony of reindeer herders in epidemic area near Pisyoto lake, herds from all foci of infection had been driven through the same place in the tundra. The thawing of permafrost provoked a landslide of a hill on the bank of the river. This could explain the finding of a single MLVA and wgSNP genotype. Unfortunately since all the transportation means in the region were being used to transport people and cargo, it was not possible to visit and sample the place (in this region roads are absent; movement is possible only by sledges with a team of reindeers or by helicopter). Two migration ways of the pathogen might be proposed: washing out of bacterial cells from deep soil layers to the ground surface, or exposure of a deep soil layer to the surface due to thawing and landslide. At the same time, the reindeers were weakened by the heat, which could have increased susceptibility to infection. Observations in the focus of the disease and a survey of veterinarians and reindeer herders gave grounds to assume that infection could occur not only by spores but also by vegetative cells. In several cases, a sick reindeer which could start recovering and stand on its legs after receiving a single dose of antibiotic, subsequently licked the muzzle of healthy animals, which fell ill and died within 24 hours.

Consequently, we cannot exclude a simultaneous spreading of infection from several isolated foci triggered by the exceptionally hot weather. A preceding large-scale epizootic, spread widely in the region and conserved in permafrost in multiple soil foci might explain the observed genetic homogeneity of the strains collected during the present outbreak. In our opinion, this alternative is not the most likely but unfortunately, strains from previous outbreaks were not preserved in collections. Therefore, there is no way to compare the strain isolated in 2016 with strains previously circulating in the region, and, accordingly, to precisely estimate the length of time during which the pathogen spores were stored in the soil. Given that the last outbreak of anthrax in Yamal was registered in 1941, it is likely that the spores remained viable in the soil for at least 75 years.

Considering the scale of the epidemic and the costs of countering it, the time interval between the beginning of reindeer disease and the beginning of antiepidemic measures may seem too large. A faster response might have allowed a better control of the epidemic. Several factors played a role in this situation:

- Presence of a large number of unvaccinated reindeers (Yamal reindeers population reaches 800 thousand), susceptible to infection and weakened by heat.
- Lack of experience in anthrax diagnosis—the last outbreak of anthrax in this region was recorded in 1941, and local veterinarians had no experience of this infection before 2016.
- Nomadic mode of cattle breeding—even a small herd can consume the limited tundra vegetation very quickly, thus breeders have to drive their herd to another place. Nomadic cattle breeding covers much larger territories than settled cattle breeding. The migration routes of different herds may cross each other. In the case of anthrax, this can rapidly increase the epidemic area, and contribute the infection to herds that migrate through the territories where sick animals were driven.

The establishment of the scale of the accident and the timely examination of the corpses was hampered by the fact that reindeer herders migrated and could not always bring dead deer for inspection. The delay in identifying patients with anthrax was also due to lack of awareness of the local population about the dangers of anthrax and about its symptoms. Local populations, even with symptoms of the cutaneous form of this disease, did not pay attention to them and considered themselves healthy. This situation arose from a set of reasons. The traditional way of life of reindeer breeders, transport and communication isolation of nomadic reindeer herders from cities is associated with a more limited access to medical care. The usually high prevalence of furunculosis prevented timely detection of anthrax affections on the skin.

All these factors favored the spreading of the infection before medical and veterinary organizations were alerted. During a hidden period, the infection may have been carried by transport (helicopters and ships), along with the goods and people who visited the outbreak, where the animals infection happened first. Unfortunately, it is not possible to retrace retrospectively the movement of people and transport between the foci of infection.

The occurrence of anthrax outbreaks after a 75-year break demonstrated that the decision to stop the vaccination was premature, in line with similar independent observations in Georgia [50]. Permafrost turned out capable to conserve viable microbial spores for a long period and to act as a reservoir of infection. Under favorable climatic conditions, these spores proved able to migrate to the surface of the soil and initiate new infection cycles.

If the Yamal strains were isolated during a large-scale epidemic, then the finding of the Yakut strains in a seemingly random place is very noteworthy. In the absence of historic record of any anthrax outbreak in this area, there was no reason to expect the finding of *B*. *anthracis* spores in the soil. The microbiological investigation was prompted by the paleontological finding of cave lions leading to the serendipitous finding of *B*. *anthracis*. Three different genotypes from soil samples taken from a depth corresponding to the upper layers of permafrost down to four meters were characterized. One genotype was very close to the Yamal genotype and belongs to the B cluster of *B*. *anthracis* whereas the two others belong to the A.Br.008/011 polytomy.

### Tentatively dating the emergence of the A.Br.008/011 polytomy

The monophyletic, strictly clonal evolution of *B*. *anthracis* implies that the whole species derives from a unique progenitor [51]. Africa is sometimes proposed as being the “cradle” of *B*. *anthracis* [52]. The strongest argument in favor of such an origin may be the discovery in Africa of additional *B*. *cereus* lineages causing an anthrax-like disease [51, 53, 54]. However, this does not help dating or locating the origin of modern lineages, even when these lineages display a strong geographic preference. A tentative dating of the Most Recent Common Ancestor (MRCA) of the *B*. *anthracis* species to 13,000–27,0000 years was previously proposed based on average mutation rate and estimates of infection cycles per year [33].

The dating of nodes along the *B*. *anthracis* phylogeny is particularly difficult and challenging because of the irregularity of its evolution due to its ecology [33, 38, 55]. In contrast with other pathogens such as *Mycobacterium tuberculosis* [42, 56, 57], branch length does not correlate with time, as previously investigated and discussed in detail [34]. Rather branch length most likely reflects the number of infection opportunities per year [33] or more rarely a mutator phenotype [37]. This number is expected to increase when *B*. *anthracis* encounters a favorable ecological niche. Each split in the tree reflects the colonization of a new ecotype allowing the fixation of an additional, independent lineage. Usually such a split will result from geographic spreading, and when a significant difference is observed in branch length, the new ecotype may be the one with the longest, faster evolving branch, reflecting the arrival in a new, naive environment. In this context, polytomies constitute exceptional opportunities to try to propose dating points. Polytomies result from the sudden colonization of multiple new ecotypes, which may reflect exceptional environmental changes. Such changes may have an anthropic origin and it may be easier to associate a polytomy with major historical events. Two large polytomies have been described so far in *B*. *anthracis* the A.Br.008/011 [38] and the derived A.Br.011/009 polytomies [43]. They constitute the “TransEurAsia” (TEA) subclade [38]. Whereas A.Br.008/011 sublineages are found across Eurasia, the six A.Br.011/009 clades are observed essentially in France and Italy [43, 44,45].

The branching of fast-evolving lineages from the same position within a unique sublineage of the A.Br.011/009 polytomy towards both West Africa and North America indicated that the contamination was exported from a geographically limited region having exchanges at the same time with both continents (Canada, Senegal-Gambia). France, seventeenth century was proposed as the most likely spatiotemporal candidate [44]. Based on the proposed dating, a tentative dating of the most recent ancestor of the A.Br.011/009 polytomy to the fourteenth or fifteenth century of the common era was further hypothesized [44].

In contrast to the A.Br.011/009 polytomy, the A.Br.008/011 polytomy is characterized by a remarkable geographic spreading. Rare, deep branching lineages are observed in Europe (Italy, Bulgaria, France) as well as Turkey, China and Yakutia (the permafrost strains, this report). There is one major event in human history prior to the sixteenth century, which could explain such a distribution, the Mongol conquests during the thirteenth century [58, 59]. Chinghis Khan assembled an empire extending from Northern China to the East side of the Caspian sea. The death of Chingis-khan in 1227 triggered the gathering of the Chingizids Armies for the election of Ögedei as new khan, and the death of Ögedei in 1241 eventually triggered a new gathering in 1246. The conquests, powered by horses, involved long-distance displacement of tens of thousands of horses. The immediate successors of Chingis-khan invaded Europe as far as Hungaria in 1237–1242 and Anatolia (Turkey) in 1241–1243. A second attempt to invade Europe took place in 1282–1283 but was a major military failure [60].

The Mongol Empire began to disintegrate in the second half of the 13th century. Despite this, the territory from China to Eastern Europe remained under the rule of Chingizids, and Eastern Europe was constantly subjected to raids from the Golden Horde for the purpose of plundering, or simply the presence of military contingents participating in wars. Thus, a common political space was established, ensuring relatively large movements of people and animals between Asia and Europe, which could further contribute to the relatively rapid and unhindered transfer of pathogens of infectious diseases between these regions. In addition to military operations, the Mongols organized a “Yam”, i.e. a chain of relay stations at certain distances to each other, allowing replacing horses for messengers or messengers themselves, and making possible to deliver cargo and documents within weeks over long distances. This postal system also could promote rapid spread of infections, but to a much lesser extent than the massive movements of armies driven by horses.

The presence of Chingizids in the European region ceased when Russian Tsar Ivan the fourth (Ivan the Terrible) conquered Western states, which were formed after the split of the Golden Horde, the Kazan Khanate in 1552, the Astrakhan Khanate in 1556 and the Siberian Khanate in 1582–1598. After that, the only post-Mongolian state remained the Crimean Khanate, which regularly carried out raids on Russia and Poland (the territories of modern Russia, Ukraine, Belarus, Lithuania, Latvia, Estonia and Moldova) until it was conquered by Catherine the Great in 1783. Despite the active raid policy of the Crimean Khanate and the constant use of the Tatar contingents by the Moscow tsars in the course of constant wars in eastern and northern Europe, the continuity of migration routes of nomads from Asia to Europe was broken.

Consequently we propose here that the root of the A.Br.008/011 polytomy corresponds to a *B*. *anthracis* ecotype present in the Mongolian armies between the first half of the 13^th^ century and the middle of the 16^th^ century. We particularly favor the 13^th^ century as the period when *B*. *anthracis* could have been transported in a short time-frame by the animals associated with the Mongolian armies, particularly war and led horses in all geographic areas covered by the deep branching lineages of the A.Br.008/011 polytomy, i.e. from Eastern Asia (China) to Eastern Europe (Hungaria, Albania, Bulgaria). After that time, we speculate that the split of the Mongolian empire would have significantly hindered the spread of an infection.

In contrast with this relatively precise dating hypothesis, we see at present no clue regarding the geographic origin of the ancestor of the A.Br.008/011 polytomy. The contamination of the Mongolian army might have occurred in many locations, from Eastern Europe to Eastern Asia.

### Apparent contradiction between the proposed dating of the emergence of the A.Br.008/011 polytomy and of the permafrost samples

Under the proposed hypothesis, the A.Br.008/011 polytomy can be conservatively dated from the early 13^th^ century to the middle of the 16th century. The deposition of the A.Br.011/008 strains including the one represented by LP51_4Ya recovered from permafrost at a depth of two and three meters would be posterior to this period. The 3Ya sublineage is even more recent, the relative length of the branch is less than half the total length of lineage “Tsiankovskii” to which it belongs.

The Yakutia permafrost soil samples were taken from alluvial (river) sediments in a wide flat valley with bayou lakes. This probably corresponds to Holocene sediments (age about 10,000 years), frozen as they accumulated. A layer of permafrost formed simultaneously in Yakutia and Yamal 20–40 thousand years ago. Radiocarbon analysis of other sediments sampled in Uyandina riverside and other places of Abyisk district at the same depth, indicated that they were 3–10 thousand years old [61]. On average, in Yakutia, the depth of seasonal thawing does not exceed 2–2.5 meters. Therefore, we expected that strains of *B*. *anthracis*, isolated from the permafrost, would be significantly older. However, the accidental nature of the exceptional paleontological finding in Yakutia, and the extraction of paleontological material and soil samples by prospectors rather than professional paleontologists or geologists, may be responsible for a weak geological information about the soil layers from which the strains were extracted. The finding of *B*. *anthracis* was quite unexpected and was triggered by the cave lions investigation, and one year passed between the excavation of the cave lions and the final soil sampling.

We also carefully examined the extent to which the present findings could be the result, or be affected, by contaminations of different kinds. However, the possibility that the strains were introduced into the soil samples under study as a result of contamination during work is most unlikely. Drift of spores from the surface is unlikely, because of the absence of reported cases at the sampling site in the past decades and in view of the absence of *B*. *anthracis* spores in the upper (minus one meter) sample. Most importantly, the initial identification of *B*. *anthracis* was made in a laboratory not working with pathogenic microorganisms and not maintaining such strain collections. The contamination in SRCAMB is also very unlikely, as it would require a simultaneous contamination with three different strains in only three among the six soil samples. In addition and most convincingly, whole genome sequence analysis of strains from the SRCAMB collection showing a similar MLVA genotype demonstrated that these are definitely different strains.

A number of conclusions can thus be robustly drawn. We looked for the presence of *B*. *anthracis* spores over a depth of six meters, going from one meter below surface down to the cave lion kittens. The state of preservation of the kittens indicated that the bodies, and the corresponding permafrost layer, remained frozen for thousands of years. Under these conditions, the most parsimonious explanation for the finding of *B*. *anthracis* strains only in the upper layers is that *B*. *anthracis* was not present in the region at the time of the death of the kittens, 5000 to 10,000 thousand years ago. Rather *B*. *anthracis* would have arrived relatively recently in three occasions represented by three distinct lineages. This diversity cannot be the result of pathogen evolution in the soil. The three genotypes are clearly positioned in different places of the *B*. *anthracis* evolutionary tree. Thus, all three strains most likely hit the ground and were conserved at different times, during various epizootics. The lack of a clear stratigraphy, that is, the isolation of isolates that represent a single genotype from soil samples from different depths, and more importantly the finding of spores lower than expected from the proposed dating would imply that microorganisms are able to migrate in permafrost. This would be compatible with current knowledge on permafrost [62, 63].

### Explaining the close relationship between the Yamal and Yakoutia *B*. *anthracis* strains

One surprising observation in our study is the very close genetic relationship of the Yamal isolates with the Yakut strain LP53_5Ya in spite of the large distance (about two thousand kilometers) separating the two locations. Despite this remoteness from each other, they are located at similar longitude, and the ecosystems located in them are almost identical. In this connection, it can be assumed that these strains are representatives of a certain "tundra" population of *B*. *anthracis*, spread on a significant territory of Northern Eurasia, at least in the past. In this case, the circulation of strains could be ensured by migration of ungulate populations, primarily reindeer.

The territory of Yakutia was inhabited by modern humans at least from the Mesolithic, but before the beginning of the 2nd millennium of the common era it was inhabited exclusively by tribes of hunters and fisher-men. The first population of herders, the ancestors of modern Yakuts, migrated here only at the beginning of the second millennium from the Baikal region. They were livestock breeders, breeding cattle and horses. In the Baikal region, they also bred sheep and camels, but it is impossible to breed them in the territory of Yakutia due to the severe climate.

The conquest by the Mongols of Southern Siberia, slightly increased the migration movement from Baikal region. Tribes with domestic reindeer that came from the south of Siberia (Baikal region), by the middle of the second millennium of the Common Era (CE) reached the territory of Western Siberia, which includes the Yamal Peninsula, and the north of Eastern Siberia, Yakutia. These tribes from the Baikal region were the ancestors of the Nenets in Western Siberia, and of the Evenks in Eastern Siberia. Theoretically, it can be assumed that in the middle of the 2nd millennium of the Common Era those and others could have been in contact in the Yenisei basin, south of Taimyr, for example, in the Turukhansk district, from where a route to the Yamal is possible. At that time reindeers were mainly used for transportation and the number of domestic deers was low. Until the 17th century there were no large herds, the maximum livestock in one farm could reach one hundred heads of deer. Large-herd reindeer herding developed only after the colonization of Siberia by Russians, beginning from the 17th and 18th centuries. Currently, the number of herds in one farm reaches several thousand heads. More knowledge on *B*. *anthracis* strains present in Northern Europe and Siberia will help better understand the history of *B*. *anthracis* spreading among reindeers.

Only in the middle of the 20th century, in connection with the beginning of mass vaccination of reindeer and the introduction of veterinary and sanitary control, obstacles arose for the free distribution of *B*. *anthracis* in the tundra zone. However, considering the ability of *B*. *anthracis* to form endospores and the presence of permafrost, capable of preserving these spores, further increasing the period of their viability, multiple soil foci could have formed by this time scattered over a vast territory. This territory is very little involved in economic activity and is extremely poorly populated. The average population density in Yakutia is 0.3 people per square kilometer with two thirds of the population living in cities, and a density in rural areas of about 0.1 person per square kilometer. This makes not only sanitation, but even detection and recording of such foci an impossible task. The events of the summer of 2016 in Yamal have shown that such foci retain their epidemiological potential for a long time, and under favorable conditions, primarily in the thawing of permafrost due to local or global warming, they can become a source of infection, causing large-scale epidemics, resulting in casualties and significant economic costs. A reactivated focus may remain active for some years. In 2017, 32 samples from the Yamal 2016 epidemic area were tested. Two samples, ash from the place of a burned deer carcass (lat. N68.24010, lon. E71.01.200) and the biological material from an incompletely burnt deer carcass (lat. N68.24989, lon. E70.44435) contained PCR detectable *B*. *anthracis* DNA. Virulent bacteria could be cultivated from both samples. The MLVA genotype was identical to the strains previously isolated in the epidemic area in 2016 [64].

If spores were able to keep viability during a year on the soil surface, then there is little doubt that during seasonal snow melting they could spread over a large area and penetrate into the soil at an unexpected depth. Thus, in the tundra areas, where at least once an anthrax outbreak was recorded, it appears necessary to continuously conduct anti-epidemic measures, such as vaccination of livestock and herdsmen, as well as maintain the readiness of medical and veterinary institutions for the diagnosis of anthrax and emergency measures for detecting the disease.

## Conclusion

In summary, we have detected three independent events of *B*. *anthracis* introduction in Northern Yakutia, stored at different depth in the permafrost. In the proposed model, the third and most recent introduction, detected at minus two meters, occurred as a side effect of Russian conquests and development of agriculture after the 17^th^-18^th^ century. The second introduction detected at minus 2 and minus 3 meters, would be the byproduct of Yakut’s population migration from Lake Baikal area after the 14^th^-15^th^ century. The first introduction detected at minus 4 meters, cannot be dated but the location in the permafrost and its genetic proximity with the strain independently found in the Yamal peninsula may indicate that it is not more than a few centuries older than the second introduction. Taking into account the previous datation proposition for the A.Br.011/009 polytomy to the 14th-15^th^ century, we propose to date the emergence of the A.Br.008/011 polytomy to the thirteenth century, in relation with the Mongolian invasions of Eastern Europe. Many more investigations will be required to evaluate the predictions of these hypotheses regarding the ecology of *B*. *anthracis* in the permafrost, as well as to know if the Mongolian Army carried *B*. *anthracis* to Eastern Europe or if they were contaminated in Eastern Europe.

## Supporting information

S1 Fig Position of the Yamal and Yakutia strains in the global *B*. *anthracis* phylogeny. Forty-two *B*. *anthracis* strains representing the main *B*. *anthracis* lineages were selected from public whole genome sequences (subset indicated in S1 Table). Red star: the tree is rooted with *B*. *cereus* strain ISSFR-23F [65]. Each circle is labelled with the corresponding strain name. The color code reflects the canSNP lineage. The very rare lineages defining the most ancient currently known splits have been found only in North America. The Yamal and Yakutia strains are arrowed. The nearest neighbors from the SRCAMB collection in terms of MLVA genotype are shown with a blue shade. The number of SNPs constituting each branch is indicated. A logarithmic scaling was used in order to visualize the shorter branches. The longest genetic distance links the MRCA of the *B*. *anthracis* species and the *B*. *cereus* outgroup. The precise position of the ancestor of the *B*. *anthracis* species along this branch is unknown.
(TIF)

S2 Fig Focus on the Yamal and Yakut lineage. wgSNP analysis was done on the four Yamal and the LP53_5Ya Yakut strain using HYU01 as outgroup. The color code is the same as in Fig 1. The use of a minimal number of closely related strains allows to maximize the number of SNPs explaining that branches are longer as compared to Fig 2.
(TIF)

S1 Table List of 1077 accession numbers comprising 1075 *B*. *anthracis* (including 11 SRCAMB strains) and two *B*. *cereus* whole genome sequence (strains JRS4 and ISSFR-23F) datasets evaluated in the present investigation (wgs datasets made publicly available before march 31 2019). The read archive or assembly accession number are indicated, together with strain name, canSNP assignment, lineage within the A.Br008/009 polytomies (when relevant), country of origin and indication of the subset of entries used in the making of each of the figures. The 11 SRCAMB strains are read archives ERR3170358 to ERR3170368. A number of archives are duplicates or redundant as indicated in the “comments” column.
(XLSX)

S2 Table MLVA profiles of described strains. (XLSX)

S3 Table LD50 values of studied strains for mice and guinea pigs. (XLSX)
