# Supplementary figures and images for: Insights from Bacillus anthracis strains isolated from permafrost in the tundra zone of Russia
Source: PLoS One. 2019 May 22;14(5):e0209140. doi: 10.1371/journal.pone.0209140 (PMC6530834; doi:10.1371/journal.pone.0209140)

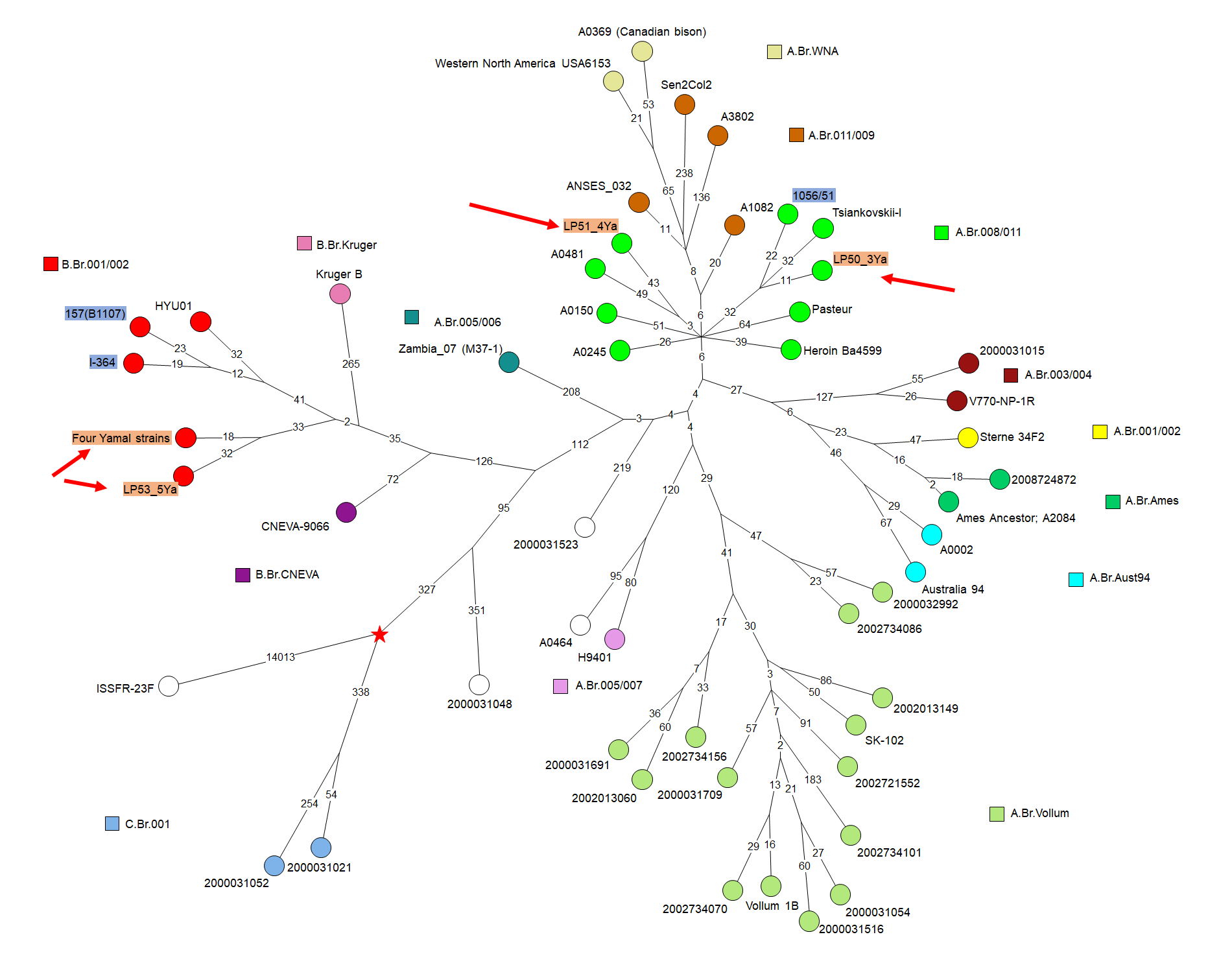

Supplement: S1 Fig — Forty-two B. anthracis strains representing the main B. anthracis lineages were selected from public whole genome sequences (subset indicated in S1 Table). Red star: the tree is rooted with B. cereus strain ISSFR-23F [65]. Each circle is labelled with the corresponding strain name. The color code reflects the canSNP lineage. The very rare lineages defining the most ancient currently known splits have been found only in North America. The Yamal and Yakutia strains are arrowed. The nearest neighbors from the SRCAMB collection in terms of MLVA genotype are shown with a blue shade. The number of SNPs constituting each branch is indicated. A logarithmic scaling was used in order to visualize the shorter branches. The longest genetic distance links the MRCA of the B. anthracis species and the B. cereus outgroup. The precise position of the ancestor of the B. anthracis species along this branch is unknown. (TIF) [file pone.0209140.s001.tif]

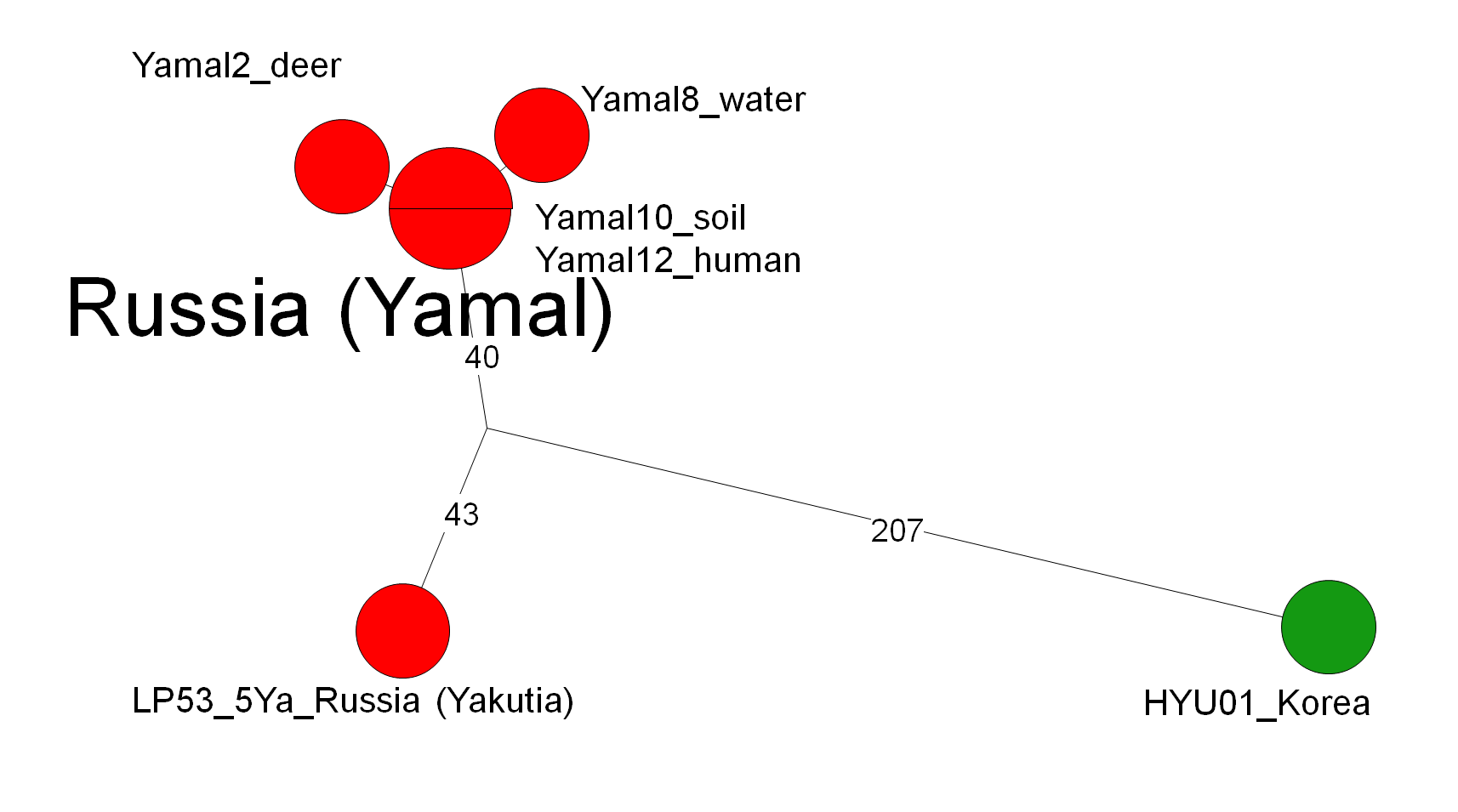

Supplement: S2 Fig — wgSNP analysis was done on the four Yamal and the LP53_5Ya Yakut strain using HYU01 as outgroup. The color code is the same as in Fig 1. The use of a minimal number of closely related strains allows to maximize the number of SNPs explaining that branches are longer as compared to Fig 2. (TIF) [file pone.0209140.s002.tif]
